# microRNAs in Ex Vivo Human Adipose Tissue Derived Mesenchymal Stromal Cells (ASC) Undergo Rapid Culture-Induced Changes in Expression, Including miR-378 which Promotes Adipogenesis

**DOI:** 10.3390/ijms21041492

**Published:** 2020-02-21

**Authors:** Megan Iminitoff, Tanvi Damani, Eloise Williams, Anna E. S. Brooks, Vaughan Feisst, Hilary M. Sheppard

**Affiliations:** 1 School of Biological Sciences, University of Auckland, 1150 Auckland, New Zealand; megan.iminitoff@gmail.com (M.I.); t.damani@auckland.ac.nz (T.D.); eloisew92@windowslive.com (E.W.); a.brooks@auckland.ac.nz (A.E.S.B.); v.feisst@auckland.ac.nz (V.F.); 2 Maurice Wilkins Centre, University of Auckland, 1150 Auckland, New Zealand

**Keywords:** human adipose-derived mesenchymal stem/stromal cells, adipose-derived stem cells, mesenchymal stem cells, microRNAs, paracrine effect, immunomagnetic bead sorting, stromal vascular fraction

## Abstract

There is clinical interest in using human adipose tissue-derived mesenchymal stromal cells (ASC) to treat a range of inflammatory and regenerative conditions. Aspects of ASC biology, including their regenerative potential and paracrine effect, are likely to be modulated, in part, by microRNAs, small RNA molecules that are embedded as regulators of gene-expression in most biological pathways. However, the effect of standard isolation and expansion protocols on microRNA expression in ASC is not well explored. Here, by using an untouched and enriched population of primary human ASC, we demonstrate that there are rapid and significant changes in microRNA expression when ASC are subjected to standard isolation and expansion methods. Functional studies focusing on miR-378 indicate that these changes in expression may have an impact on phenotype and function. Specifically, we found that increased levels of miR-378 significantly promoted adipogenesis in late passage ASC. These results are informative to maximizing the potential of ASC for use in various clinical applications, and they have implications for targeting microRNAs as a therapeutic strategy for obesity or metabolic disease.

## 1. Introduction

Human adipose tissue derived mesenchymal stromal cells (ASC) are showing promise in the clinic for a range of inflammatory and degenerative conditions [1]. These cells are derived from the heterogeneous stromal vascular fraction (SVF), which is isolated from the lipoaspirate of patients. The ex vivo ASC cell surface phenotype was defined as CD45-CD235a-CD31-CD34+CD13+CD73+CD90+CD105+ by the International Society for Cell and Gene Therapy (ISCT) and International Federation for Adipose Therapeutics and Science (IFATS) in 2013 [2]. ASC are typically ‘purified’ and expanded from the SVF via adherent cell culture, where they retain a similar cell-surface expression to ex vivo cells but CD34+ expression is lost. Adherent, fibroblastic cells that also display ‘trilineage potential’, in that they are able to differentiate into adipocytes, osteoblasts, and chondrocytes *in vitro*, are defined as ASC.

We previously reported a method that allowed for the rapid enrichment of a defined and untouched ex vivo ASC population that were then compared to culture-derived ASC [3]. This comparison found that ex vivo ASC demonstrate rapid changes in mRNA expression when exposed to adherent-tissue culture that are likely to effect the function and potency of these cells. Here, we use the same enrichment process to examine the effect that time in tissue culture has on microRNA expression. As far as we are aware, this has not been previously examined in untouched and uncultured ex vivo ASC, or indeed in any uncultured cell type. microRNAs are a class of small RNAs that have a critical role to play in many cellular processes, as they are powerful regulators of gene-expression [4]. Increasingly, it is becoming apparent that ASC exert their therapeutic effect via a paracrine mechanism [5,6]. Although the exact mode of action is not yet fully elucidated, it is likely to involve secreted proteins and/or nucleic-acid based materials that may be embedded in extra-cellular vesicles. Shuttling of select-miRNAs in microvesicles and delivery to target cells has been observed in human bone-marrow MSCs [7]. A similar phenomenon was also recently reported in ASC, where exosome-shuttled miRNAs were found to reduce the expression of pro-inflammatory cytokines and chemokines in an in vitro model of chronic inflammation [8]. Therefore, the therapeutic potency of ASC is likely to be linked in part to microRNA expression [5]. While using microarray profiling, here we report that global microRNA expression patterns change rapidly and significantly within days of exposure to adherent tissue culture. Therefore, this modulation might affect their paracrine effect and clinical utility.

microRNAs (miRNAs) are also important regulators of cellular function [9] and they have been shown to be involved in ASC differentiation [10]. The role of microRNAs in promoting lineage-specific differentiation in ASC has been relatively well explored [11,12]. However, the role of microRNAs in maintaining non-differentiated ASC is less well understood. We previously observed that ex vivo ASC contain a greater proportion of cells with activity in in vitro differentiation assays when compared to culture-purified ASC. We hypothesised that the modulation of global microRNA expression in cultured ASC might, in part, account for the loss of activity in in vitro differentiation assays. We also hypothesised that knowledge of these changes could be used as a novel option to direct cell fate and activity. Experiments with miR-378, which is significantly down regulated after 28 days in culture, supported this hypothesis. We show that activity in an in vitro adipogenic assay can be significantly enhanced in late passage ASCs when this microRNA is over-expressed. microRNAs are known to have pleiotropic effects, as individual microRNAs can potentially target many genes. Therefore, it is likely that culture-induced changes in microRNA expression will significantly affect the clinical potential of ASC. Our findings are particularly informative to ASC research, which demonstrates variability in cell isolation and expansion protocols.

## 2. Results

We previously reported on culture-induced mRNA changes in ex vivo ASC [3]. We were also interested to examine what effect time in culture would have on microRNA expression, as microRNAs are critical regulators of cellular function, including metabolism, homeostasis, proliferation, and cell fate [9]. To this end, we used immunomagnetic bead sorting to isolate *ex vivo*, untouched ASC from three donors while using a cocktail of FITC-labelled antibodies (anti-CD31, CD45, CD146, and CD235a) designed to remove all non-ASC from the SVF, as described previously [3]. Flow cytometry analyses indicated that the post sort purity of the enriched fraction was > 97% for CD34+, CD73+, and CD90+ (data not shown). These ex vivo ASC (herein referred to as ex vivo or MACS-derived ASC, were subjected to a quantitative adipogenic differentiation assay that assesses FABP4 expression by immunohistochemistry. In agreement with our previous report [3], a significantly greater proportion of ex vivo ASC expressed FABP4 in this assay when compared to ex vivo ASC which were cultured for 28 days (day 0 = 57.3%, st. dev. +/− 21.6% versus day 28 = 28.5%, st. dev. +/− 14.9%, *p* value = 0.03, data not shown). These ex vivo ASC were cultured in standard tissue culture conditions for 0, 3, 28 days (passage 4), or approximately 63 days (passage 9), after which total RNA, including the small RNA species, was isolated in order to examine the expression of microRNAs in these cell populations. These time-points were chosen to examine microRNA expression in uncultured (day 0) or minimally cultured cells (day 3). The 28 day time-point was chosen to match the time point used to enrich for culture-derived ASC from SVF. We have previously demonstrated that 28 days was sufficient to achieve homogeneity of ASC that was based on CD34, CD73, and CD90 [13]. In addition, in general, three to four passages is accepted as a pure population [14], and is comparable to standard methods used to enrich for ASC. We further included a passage nine time-point to assess what affect extensive time in culture (> 60 days) might have on microRNA expression. RNA was subjected to microarray analysis using Affymetrix GeneChip microRNA 3.0 Arrays. Quality controlled and robust microarray average (RMA) normalised data was further analysed while using Affymetrix transcriptome analysis console software to identify any microRNAs that were differentially expressed between the treatment groups.

1733 microRNAs were interrogated using the microarray platform. Of these, 49 microRNAs were down regulated more than two-fold and 21 microRNAs were up regulated when comparing day 0 to day 3 data as averaged over the three donors (Supplementary Table S1). These data suggest that there are rapid (within 72 h or less), major, and significant culture-induced changes in microRNA expression. Ninety-six microRNAs were identified as down regulated more than two-fold and 35 up regulated when comparing day 0 to day 28 cultured cells (Supplementary Table S1). See Table 1 for a list of all microRNAs with a fold change >10 when comparing microRNAs that were expressed in ex vivo ASC (day 0) to ex vivo ASC that had then been cultured in standard conditions for 3 (day 3) and 28 (day 28) days (*p* value > 0.05).

**Figure 1:**
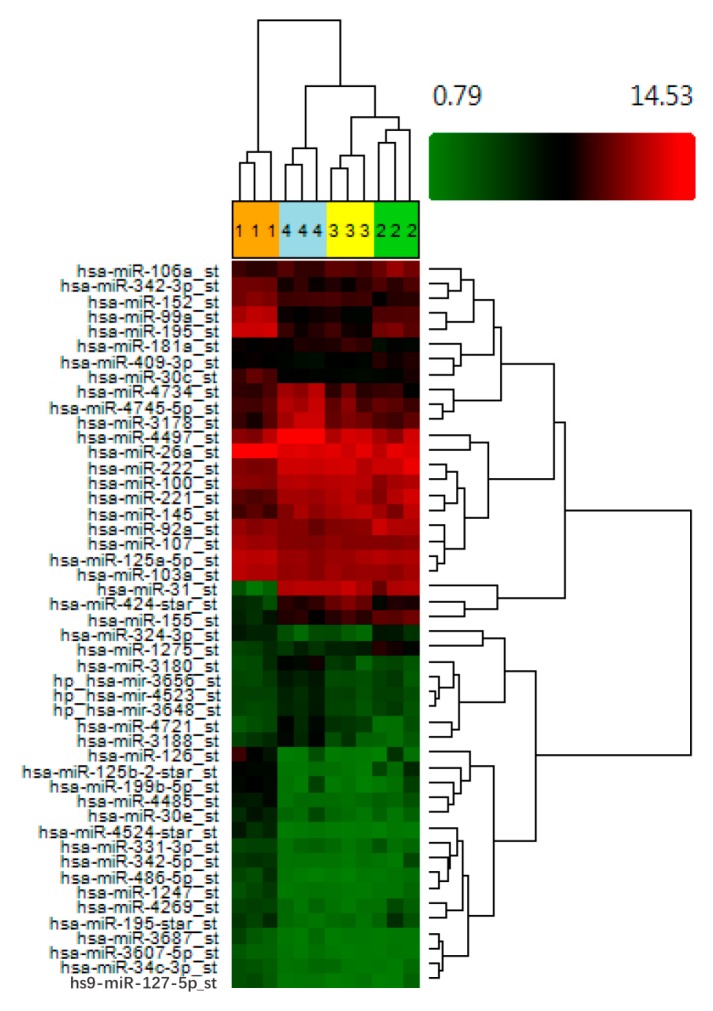
Compared to ex vivo ASC, cultured ASC exhibit a more similar microRNA expression profile regardless of the time spent in standard tissue culture conditions. MACS-derived ASC from three different donors were subjected to standard tissue culture conditions and microarray analysis was performed at day 0 (group 1 shown in orange), day 3 (group 2 shown in green), day 28 (group 3 shown in yellow), and day 63 (group 4 shown in blue) post sort. A heatmap and dendogram was generated using Transcription Analysis Console™ software (Affymetrix) and a list of all transcripts which exhibited a fold change > 2 (across all four conditions, with an ANOVA *p* value < 0.005). Green represents downregulated genes and red up regulateded genes from three donor samples.

When comparing day 3 to day 28 data, 10 microRNAs were down regulated more than two-fold and 10 microRNAs were up regulated (Supplementary Table S1). Finally a comparison of day 28 (passage 4) and day 63 (passage 9) data indicates that changes in microRNA expression continue with 76 microRNAs found to be up regulated and 10 microRNA downregulated more than two-fold (albeit fold changes in expression were more modest when comparing these time-points—see Supplementary Table S1). This indicates that there are on-going changes in microRNA expression with time spent in culture. The dendogram that is shown in Figure 1 represents the results of hierarchical clustering of all microRNAs with an expression level > 2.0 (with an ANOVA *p* value < 0.005 across all four conditions). This groups uncultured cells together and all cultured cell populations as a separate group, regardless of the time spent in culture. 

**Figure 2:**
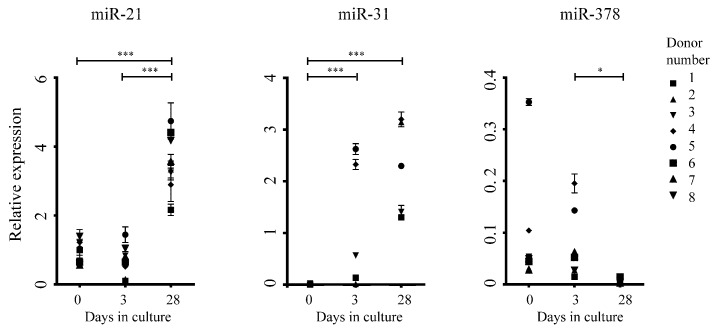
Quantitative RT-PCR validates microarray results. qRT-PCR was used to validate a subset of the microarray data (target microRNAs miR-21, miR-31, and miR-378 as indicated) in eight subsequent donors (D1–D8). Error bars represent technical repetitions. * denotes *p* value < 0.05, *** denotes *p* value < 0.001.

The expression levels of miR-21, miR-31, and miR-378 (which according to the microarray data were up regulated 20-fold, 721-fold or down regulated 36-fold, respectively, when comparing ex vivo ASC (day 0) to ex vivo ASC cultured for 28 days) were validated by quantitative real-time PCR (qRT-PCR). The qRT-PCR results from ex vivo ASC and ex vivo ASC, which were then cultured for three or 28 days in standard tissue culture conditions derived from eight new donors are shown in Figure 2. In each case, there was a statistically significant up or down regulation in microRNA expression that validated the expression trends that were observed in the microarray data. 

**Figure 3:**
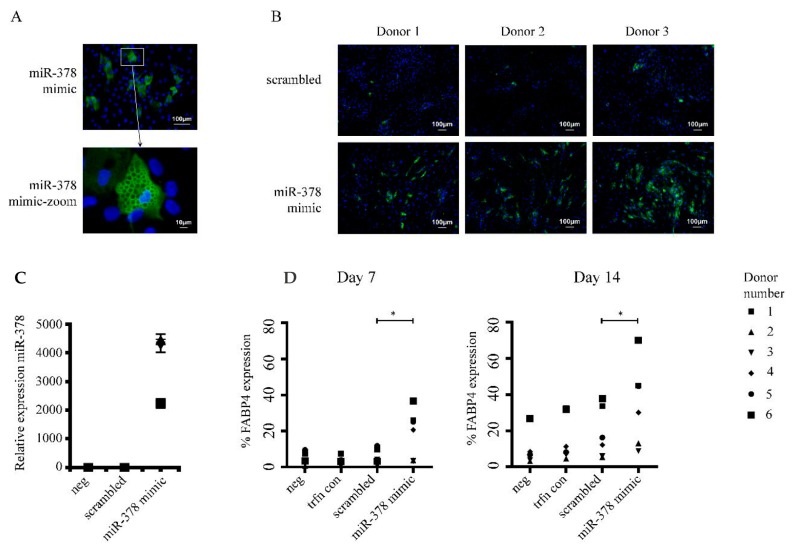
Over-expression of miR-378 increases the proportion of ASC that express FABP4 in an adipogenic assay. Late passage ASC were transfected with a miR-378 mimic and subjected to an in vitro adipogenic differentiation assay. Panel (**A**) shows a representative image of ASC subjected to the adipogenic differentiation assay taken at 20 X magnification. A single lipid-droplet containing cell staining positive for FABP4 expression is shown below in a cropped and enlarged image (labelled ‘zoom’). FABP4 positive cells are stained green and nuclei are stain blue. Panel (**B**) shows representative images from the adipogenic differentiation assay taken at day seven for quantification purposes for three donors (D1–D3) transfected with the miR-378 mimic or a scrambled control. The graph in (**C**) represents the relative expression of miR-378 in cells one week post transfection. Panel (**D**) shows the percentage of cells that stained positive for FABP4 expression after quantification at day 7 and 14 of the adipogeneic assay, *n* = 6. * denotes *p* value < 0.05.

We were next interested to test whether any of the observed changes in microRNA expression might be functionally important. Our hypothesis was that the culture-induced changes in microRNA expression could affect cell fate and might underlie the loss of activity that we observed in in vitro differentiation assays. Taking a reductionist approach, miR-378 was selected for over-expression studies in late passage ASC (cells between passage 18–30) that had been shown to express low levels of this microRNA when compared to ex vivo ASC or ex vivo ASC cultured for three days (Figure 2). The transfection rates were first optimized while using a BLOCK-iT™ Fluorescent Oligo (Invitrogen) that indicated that transfection rates after seven days were routinely > 90% (data not shown). Next, a miR-378 mimic or a scrambled control were transfected into late passage ASC which were then subjected to an adipogenic assay (see Figure 3A) with FABP4 expression assessed at day 7 and day 14. The over-expression of miR-378 was confirmed by qRT-PCR (see Figure 3C) and based on their chemical similarities an equal uptake of the scrambled control was assumed. Figure 3B shows representative images taken for quantification of FABP4 expression from three donors at day 7 of the adipogenic assay. The quantification of FABP4 expression at day 7 and day 14 of the adipogenic assay are represented by the graphs in Figure 3D. This data indicates that transfection with a miR-378 mimic significantly increased the percentage of cells expressing FABP4 compared to the scrambled control at both time points. Typically low levels of FABP4 expression are observed in this assay at day 7 in late passage cells, so these data also suggest that miR-378 expression accelerates the accumulation of FABP4. Therefore, the loss of miR-378 in cultured ex vivo ASC is likely contributing to an altered cellular phenotype and reduced activity in adipogeneic assay.

## 3. Discussion

The unique enrichment process that was used to isolate an untouched population of ex vivo ASC has allowed us to examine culture-induced changes in microRNA expression. As far as we aware this is the first report of its kind using uncultured cells as a point of comparison. microRNAs are important players in stem cell fate and they are likely to be involved in the paracrine mechanism of clinical utility of ASC. Therefore, the marked culture-induced changes in microRNA expression that we observe will impact cellular function and they are likely to alter the clinical benefits that are conferred by these cells. Understanding these changes will be informative in developing optimum protocols for isolating ASC populations for specific clinical applications. Although microRNA levels could be manipulated artificially by using mimics or inhibitors, altering endogenous expression levels by altering the length of time in culture, or avoiding cell-culture altogether, would present a more straight forward and clinically translatable approach.

Our hypothesis was that changes in microRNA expression may underlie the loss of activity that was observed in in vitro differentiation assays that we and others had observed [3,15,16,17]. The data that are presented here indicate that that is the case, as demonstrated by an over-expression of miR-378 leading to an increase in FABP4 accumulation, which is suggestive of increased differentiation down the adipogenic pathway. miR-378 has been previously shown to play a role in adipogenesis. Knock-out mice for miR-378/miR378* exhibited reduced adipocyte size when compared to their wild-type littermates [18] and transgenic mice over-expressing miR-378 demonstrated an increased mass in brown adipose tissue [19]. The up regulation of miR-378 was observed during adipogenesis in a bone-marrow derived mesenchymal cell line (ST2 cells) [20]. In addition, the upregulation of miR-378 in brown adipose tissue has been reported in murine pre-adipocyte cells [21] and tissues [19]. However, as far as we are aware, this is the first demonstration of miR-378 over-expression correlating with increased FABP4 expression, indicative of adipogenesis, in primary, human ASC. Our adipogeneic assay does not discriminate between white, beige, or brown adipocytes, but, as there is a demonstrated link between miR-378 and brown adipose tissue, it may be speculated that the over-expression of this microRNA is driving the differentiation of beige or brown fat. Therefore, targeting this microRNA in vivo could be a strategy for expanding brown adipose tissue in humans as a therapeutic strategy for obesity or metabolic disease. 

The possibility remains that, in addition to enhancing adipogenesis, this microRNA might also increase other aspects of cellular potency. For example, when miR-378 was over-expressed in mouse bone-marrow derived stem cells, they exhibited increased proliferation rates and decreased rates of apoptosis [22]. A similar phenotype was also observed when miR-378 was overexpressed in chronic myeloid leukemia K562 cells [23]. The over-expression of miR-378 in a human primary glioblastoma cell line promoted a stem cell phenotype with an increase in proliferation, adipogeneic differentiation, and colony formation [24]. These observations align with the loss in proliferation potential (see Supplemental Table S2) and corresponding loss in miR-378 expression that we observe as ASC are cultured over time.

The most differentially expressed microRNA when comparing ex vivo to cultured ex vivo ASC was miR-31 (with an 721-fold increase in expression when comparing day 0 to day 28). This has been described as a potential general inhibitor of differentiation [25], as it has been shown to inhibit adipogenesis in a murine mesenchymal stem cell line C3H10T1/2 [26] and to inhibit osteogenesis in human bone-marrow mesenchymal stem cells [27,28,29]. Similarly, miR-138 (with a 106-fold increase in expression when comparing day 0 to day 28) had also been shown to inhibit adipogenesis [30] and osteogenesis [31]. Therefore, it is possible that an increased expression of these microRNAs in combination with reduced expression of miR-378 is contributing to the loss of activity over time in in vitro differentiation assays.

It is likely that microRNA secretion in microvesicles will also be altered with time in culture, due to the marked differential expression in microRNAs, although we note that selective sorting of microRNAs into microvesicles can occur [32]. For some applications, such as enhancing angiogenesis for ischemic disease, cultured (and expanded) ASC might provide an enhanced therapeutic potential compared to ex vivo ASC. For example, miR-31 and miR-21 are more highly expressed in cultured ASC as compared to ex vivo ASC. Microvesicle transport of miR-31 from ASC to endothelial cells has been shown to enhance angiogenesis [33]. It has also been recently shown that exosomes derived from murine ASC over-expressing miR-21 led to enhanced vascularisation of an endothelial cell line in vitro [34]. However, further studies are required to accurately determine the microRNA profile that is present in microvesicles derived from ex vivo ASC.

Our data reveal the marked and rapid culture-induced changes in microRNA expression that occur when ex vivo ASC are subjected to standard tissue culture. Although these cells represent a defined and enriched population, we have recently shown that they still retain a high level of heterogeneity [3]. Whether the changes in expression we observe reflect changes occurring across the whole population of cells or within specific subsets remains to be determined. Therefore, further studies are warranted once this heterogeneity has been unraveled.

## 4. Materials and Methods

### 4.1. Processing Lipoaspirate

Lipoaspirate was obtained from informed healthy, non-obese, female donors undergoing elective liposuction with protocols that were approved by the Northern A Health and Disability Ethics Committee (approval number NTX/07/02/003). One litre of lipoaspirate was washed twice with an equal volume of phosphate-buffered saline (PBS) and digested with 0.15% Collagenase type I (Life Technologies, Carlsbad, CA, USA) in PBS for 60 min. at 37 °C with occasional mixing. Cells were pelleted by centrifugation at 1800 rpm for 10 min. resulting in the Stromal Vascular Fraction (SVF). The pellet was resuspended in 50 mL pre-warmed ASC medium (Dulbecco’s modified eagle media/ Ham’s F12 nutrient mixture (DMEM F-12; Life Technologies, Carlsbad, CA, USA) supplemented with 10% Fetal Bovine Serum (FBS), (Life Technologies, Auckland, NZ), 1% Penicillin-Streptomycin 10,000 U/mL (Invitrogen, Carlsbad, CA, USA) and 1 × GlutaMAX (Invitrogen, Carlsbad, CA, USA) and then passed through a 100 μm Falcon™ cell strainer (BD). SVF was pelleted again and resuspended in 50% ASC media and 50% freezing media (FBS plus 20% DMSO, Sigma–Aldrich, St. Louis, MO, USA) and cryopreserved in liquid nitrogen. From eight donors the average SVF yield from 1 litre of lipoaspirate was 3 × 10^8^ cells (data not shown).

### 4.2. Culturing SVF to Isolate Culture-Derived ASC

Frozen SVF suspensions were thawed and for each donor vials were split to allocate half for MACS sorting and half for plastic adherent culture. On average, 5 × 10^6^ cells were plated into a Falcon™ T75 tissue culture flask in ASC medium (see above). When the cells reached 90% confluency, they were passaged, typically once a week, for 28 days (four passages). Cell purity was assessed by flow cytometry, as described previously [3]. 

### 4.3. MACS Sorting Cells to Isolate MACS-Derived ASC

Frozen SVF suspensions were thawed and, for each donor, vials were split to allocate half for MACS sorting and half for plastic adherent culture. Both of the isolation methods were conducted in parallel and cells from each donor were cultured separately. ASC were enriched while using MACS™ anti-FITC microbeads (Miltenyi, Bergisch Gladbach, Germany), as described previously [3]. The antibody cocktail consisted of 2.5 µls of each of the following anti-human FITC-conjugated antibodies: CD31 (clone MW59), CD45 (clone HI30), CD146a (PIH12), and CD235a (clone H1246) (all from BioLegend, San Diego, CA, USA). In brief, the SVF single cell suspension was pelleted and resuspended in 100 μL MACS buffer per 10^7^ cells and incubated with the antibody cocktail on ice for 10 min. protected from light. The cells were washed twice with MACS buffer and resuspended in 90 μL of buffer per 10^7^ cells. Cells were then incubated with 10 μL of anti-FITC MACS microbeads (Miltenyi, Bergisch Gladbach, Germany) per 10^7^ cells for 15 min. at 4 °C and washed with MACS buffer. These were resuspended in 500 μL of buffer and then applied to a pre-cooled LS column (Miltenyi, Bergisch Gladbach, Germany). Post sort purity was assessed by flow cytometry, as described previously [3].

### 4.4. Adipogenic Differentiation Assays

Adipogenic staining was performed, as described previously [35]. In brief, the cells were plated in a 96-well late with ASC media (DMEM/F12 media (Life Technologies, Carlsbad, CA, USA) that was supplemented with 10% FBS, 1% GlutaMax (Life Technologies, Carlsbad, CA, USA) and 1% penicillin/streptomycin (Life Technologies, Carlsbad, CA, USA) and cultured at 37 °C, 5% CO_2_. On day four, 100 μL of media was replaced with adipogenic differentiation media (ASC media with 1 µM dexamethasone, 10 µM insulin, 0.5 mM 3-isobutyl-1-methylxanthine (IBMX), and 200 µM indomethacin (all from Sigma Aldrich, St. Louis, MO, USA) and standard ASC media was added to control wells. Half media changes were performed every three days until day 14. The cells were then subjected to immunocytochemistry while using a 1:200 dilution of rabbit anti-human FABP4 polyclonal antibody (Cat #10004944, Cayman Chemicals, Ann Arbour, MI, USA) and then incubated with a 1:200 dilution Alexa Fluor^®^ 488 conjugated goat anti-rabbit IgG secondary antibody (Cat # A11008, Molecular Probes^®^, Eugene, OR, USA) and 1:2000 diluted DAPI (Cat# D3571, Molecular Probes^®^, Eugene, OR, USA). For quantification purposes, fluorescent images were taken while using the ImageXpress Micro XLS high content screening system (Molecular Devices™, San Jose, CA, USA). Nine images were taken per well at 10 × magnification and quantitative data were generated while using the MetaXpress v 5.3.0.1 (Molecular Devices™, San Jose, CA, USA) software. The qualitative images were acquired at room temperature while using 20x/0.50 numerical aperture Leica objectives, a SPOT camera (SPOT Imaging, Sterling Heights, MI, USA), and analySIS FIVE software (Olympus, Tokyo, Japan). Images and figures were generated using Cytosketch (available online: http://www.cytocode.com/).

### 4.5. Microarrays

The cells were FACS sorted on a BD SORP FACS Aria II using the same antibody cocktail as described for the MACS sort, and post-sort analysis was as above. Cells were washed once in ice-cold PBS and total RNA was purified while using the miRVANA kit (Ambion, Austin, Tex, USA). RNA integrity was assessed using a Bioanalyser (Agilent, Santa Clara, CA, USA). 100 ng of RNA were reverse transcribed and labelled using the Genechip 3′ IVT Express kit and hybridised to GeneChip miRNA v3.0 Arrays (Affymetrix, Santa Clara, CA, USA), according to the manufacturer’s protocols. The fluorescent signals were recorded by an Affymetrix scanner 3000 using Gene Chip Operating Software. The Affymetrix^®^ Expression Console™ Software was used to carry out quality control analysis. Affymetrix^®^ Transcriptome Analysis Console (TAC) 3.0 (Santa Clara, CA, USA) was used to determine microRNAs that were differentially expressed between different conditions (day 0, day 3, and day 28). Only those genes that showed an ANOVA *p*-value of less than 0.05 and a fold difference between > 2 and < −2 were considered as being differentially expressed between the conditions. The *p*-value and fold differences were an average obtained from three different donors. Microarray data have been deposited in the Gene Expression Omnibus (GEO) database and can be accessed via accession no. GSE142429.

### 4.6. RT-PCR

RT-PCR was performed, as described previously [36]. In brief total RNA was isolated from all samples while using a miRVANA kit (Ambion, Austin, Tex, USA). First-strand cDNA was synthesized using the TaqMan^®^ microRNA Reverse Transcription Kit (ThermoFisher Scientific, Waltham, Mass, USA). Quantitative real-time (RT)-PCR was carried out on a 7900HT Real Time PCR System (Applied Biosystems, Foster City, CA, USA) using TaqMan^®^ FAST Universal PCR Master Mix (Roche, Basel, Switzerland), gene specific TaqMan^®^ probes and between 2–10 ngs cDNA per reaction. The PCR cycling parameters were 20 s at 95 °C and then 40 cycles of 1 s at 95 °C, followed by 20 s at 60 °C. The results were normalised against RNU44. 

### 4.7. Transfection of Late Passage ASC

Transfection experiments were optimised while using the BLOCK-iT™ Fluorescent Oligo (Invitrogen, Carlsbad, CA, USA). Cells were plated at 1 × 10^4^ cells per well in a 96-well plate in 200μL of antibiotic-free standard ASC media (described above). They were transfected with 0.5 ul Dharmafect 1 (GE Dharmacon, Lafayette, CO, USA) with 0.8 uls of a 5 uM solution of MC11360 human miR-378-3p mimic (ThermoFischer Scientific, Waltham, Mass, USA) or mirVana™ miRNA scrambled Negative Control #1 (ThermoFischer Scientific, Waltham, Mass, USA) the next day (day 1) and at day 8. After 24 h media, these cells were subjected to a standard adipogenic differentiation assay, as described above.

### 4.8. Statistical Analysis

Unless otherwise stated, statistical analysis was performed while using Microsoft Excel software. Significance was assessed using two-tailed, type 1 t-tests with *p* values < 0.05 being considered to be significant. * denotes a *p* value < 0.05, ** denotes a *p* value < 0.01, and *** denotes a *p* value < 0.001. 

## Figures and Tables

**Table 1:** There are significant changes in the expression of microRNAs when ex vivo adipose tissue-derived mesenchymal stromal cells (ASC) are subjected to standard tissue culture.

	Day 0 Versus Day 3				Day 0 Versus Day 28		
Down	FC	Up	FC	Down	FC	Up	FC
miR-146b-5p	60.7	miR-31	678.0	miR-126	82.7	miR-31	720.9
miR-126	54.1	miR-4521	84.7	miR-146b-5p	57.2	miR-138	106.1
miR-148a	43.9	miR-3613-3p	19.8	miR-199b-5p	52.8	miR-4521	56.1
miR-199b-5p	42.6	miR-155	19.1	miR-125b-2-star	48.5	miR-424-star	29.6
miR-224-star	31.0	miR-1275	16.4	miR-378	36.1	miR-210	29.4
miR-337-5p	30.7	miR-1972	15.6	miR-148a	33.9	miR-503	25.2
miR-4524-star	20.2	miR-424-star	10.0	miR-328	28.3	miR-21	20.2
miR-140-5p	18.6			miR-378c	25.5	miR-3613-3p	17.2
miR-411	14.6			miR-99a	23.9	miR-493	17.1
miR-15a	12.3			miR-497	22.1	miR-31-star	17.1
miR-497	12.2			miR-4485	20.3	miR-125b-1-star	16.4
miR-4485	11.8			miR-4524-star	20.2		
				miR-195	18.9		
				miR-30e	14.4		
				miR-26b	13.1		
				miR-148b	11.3		
				let-7d-star	11.1		
				miR-572	10.7		

Transcription Analysis Console™ software (Affymetrix, Santa Clara, CA, USA) was used to analyse the fold changes (FC) in miRNA microarray data derived from ex vivo ASC (day 0) or ex vivo ASC that had been cultured from three (day 3) or 28 (day 28) days. Down and up regulated miRNAs with a fold change >10 and an ANOVA *p* value > 0.05 are shown. For a full list of all microRNAs with a fold change of > 2 see Supplemental Table S1.

## Supplementary Materials

Supplementary materials can be found at https://www.mdpi.com/1422-0067/21/4/1492/s1.
