# Supplementary material for: microRNAs in Ex Vivo Human Adipose Tissue Derived Mesenchymal Stromal Cells (ASC) Undergo Rapid Culture-Induced Changes in Expression, Including miR-378 which Promotes Adipogenesis
Source: Int J Mol Sci. 2020 Feb 21;21(4):1492. doi: 10.3390/ijms21041492 (PMC7073112; doi:10.3390/ijms21041492)
Supplement: Supplementary file 1 [file ijms-21-01492-s001.pdf]

Supplemental Table S1

Fold changes in microRNAexpression when comparing *ex vivo* ASC (day 0) to *ex vivo* ASC cultured for 3, 28 or 63 days (fold change >2, ANOVA p value < 0.05)

Data derived from Transcription Analysis Console software (Affymetrix).

Day 0 versus day 3
Down regulated miRNAs

**Transcript ID**	**Fold Change**	**ANOVA p-value**
hsa-miR-146b-5p	60.7	0.012268
hsa-miR-126	54.13	0.026306
hsa-miR-148a	43.9	0.019138
hsa-miR-199b-5p	42.62	0.008064
hsa-miR-224-star	31	0.02797
hsa-miR-337-5p	30.67	0.018862
hsa-miR-4524-star	20.22	0.000363
hsa-miR-140-5p	18.6	0.036066
hsa-miR-411	14.56	0.025582
hsa-miR-15a	12.27	0.032517
hsa-miR-497	12.15	0.002446
hsa-miR-4485	11.77	0.001032
hsa-miR-342-5p	9.81	0.036402
hsa-miR-125b-2-star	9.75	0.029357
hsa-miR-660	9.31	0.002604
hsa-miR-30e	9.3	0.001772
hsa-miR-409-5p	8.48	0.03751
hsa-miR-371b-5p	8.33	0.042127
hsa-let-7d-star	7.5	0.013373
hsa-miR-26b	6.7	0.021077
hsa-miR-486-5p	5.57	0.007324

Day 0 versus day 3
Up regulated miRNAs

**Transcript ID**	**Fold Change**	**ANOVA p-value**
hsa-miR-31	678	0.000426
hsa-miR-4521	84.67	0.000268
hsa-miR-3613-3p	19.82	0.013408
hsa-miR-155	19.11	0.000684
hsa-miR-1275	16.42	0.004977
hsa-miR-1972	15.61	0.044059
hsa-miR-424-star	10.02	0.013439
hsa-miR-4298	6.21	0.035325
hsa-miR-4728-5p	4.76	0.032584
hsa-miR-23a-star	4.75	0.014396
hsa-miR-145	4.31	0.021033
hsa-miR-3141	4.25	0.03013
hsa-miR-221	3.87	0.023817
hsa-miR-17	3.73	0.011641
hsa-miR-4749-5p	3.44	0.021247
hsa-miR-222	3.35	0.002099
hsa-miR-93	3.05	0.023782
hsa-miR-106a	2.8	0.008286
hsa-miR-4459	2.79	0.044308
hsa-miR-20a	2.48	0.025748
hsa-miR-1207-5p	2.43	0.01798

hsa-miR-34c-5p	5.56	0.03188
hsa-miR-3188	5.19	0.009308
hsa-miR-127-5p	5.06	0.027944
hsa-miR-1247	5.02	0.008226
hsa-miR-99a	4.76	0.000304
hsa-miR-30c	4.55	0.018548
hsa-miR-195	4.49	0.001025
hsa-miR-331-3p	3.77	0.017661
hsa-miR-3609	3.71	0.017366
hsa-miR-194	3.69	0.031226
hsa-miR-4449	3.67	0.043727
hsa-miR-152	3.51	0.00306
hsa-miR-887	3.47	0.039711
hsa-miR-34c-3p	3.22	0.012492
hsa-miR-30d	3.15	0.044579
hsa-miR-3687	3.12	0.021352
hsa-miR-16	2.92	0.021574
hsa-miR-1281	2.8	0.048139
hsa-miR-140-3p	2.56	0.001312
hsa-miR-22	2.54	0.02307
hsa-miR-130a	2.53	0.030426
hsa-miR-26a	2.44	0.012195
hsa-miR-664-star	2.27	0.005784
hsa-miR-628-3p	2.2	0.002515
hsa-miR-181a-2-star	2.17	0.019858
hsa-miR-361-5p	2.1	0.007598
hsa-miR-181a	2.09	0.022917
hsa-miR-3607-5p	2.05	0.004245

Day 0 versus day 28
Down regulated miRNAs

Day 0 versus day 28
Up regulated miRNAs

Transcript ID	Fold Change	ANOVA p-value	Transcript ID	Fold Change	ANOVA p-value
hsa-mi126	82.71	0.001736	hsa-miR-31	720.93	0.000108
hsa-miR-146b-5p	57.19	0.001715	hsa-miR-138	106.1	0.000765
hsa-miR-199b-5p	52.83	0.000053	hsa-miR-4521	56.05	0.001809
hsa-miR-125b-2-star	48.48	0.000024	hsa-miR-424-star	29.63	0.000919
hsa-miR-378	36.13	0.003933	hsa-miR-210	29.41	0.003326
hsa-miR-148a	33.87	0.008751	hsa-miR-503	25.22	0.00018
hsa-miR-328	28.27	0.000116	hsa-miR-21	20.2	0.014756
hsa-miR-378c	25.46	0.004495	hsa-miR-3613-3p	17.23	0.035875
hsa-miR-99a	23.87	0.000745	hsa-miR-493	17.14	0.036988
hsa-miR-497	22.08	0.011981	hsa-miR-31-star	17.1	0.008346
hsa-miR-4485	20.34	0.000102	hsa-miR-125b-1-star	16.35	0.000776
hsa-miR-4524-star	20.18	0.000317	hsa-miR-708	9.82	0.006189
hsa-miR-195	18.89	0.000476	hsa-miR-224	8.92	0.016623
hsa-miR-30e	14.37	0.000555	hsa-miR-155	8.18	0.002164
hsa-miR-26b	13.11	0.006179	hsa-miR-181a-2-star	8.09	0.000797
hsa-miR-148b	11.28	0.03691	hsa-miR-145	6.36	0.001088
hsa-let-7d-star	11.14	0.000274	hsa-miR-181b	5.87	0.016689
hsa-miR-572	10.69	0.003981	hsa-miR-138-1-star	5.66	0.013535
hsa-miR-660	9.48	0.008845	hsa-miR-424	5.41	0.000105
hsa-miR-421	9.36	0.005748	hsa-miR-143	5.21	0.002385
hsa-miR-1180	9.34	0.03546	hsa-miR-4454	4.39	0.01616
hsa-miR-409-5p	9.04	0.0396	hsa-miR-222	4	0.002773
hsa-miR-378f	9	0.006276	hsa-miR-221	3.69	0.000646
hsa-miR-342-5p	8.93	0.000087	hsa-miR-2277-5p	3.69	0.026393
hsa-miR-146a	7.8	0.003716	hsa-miR-4669	3.42	0.000188
hsa-miR-486-5p	7.49	0.003856	hsa-miR-3911	3.33	0.04767
hsa-miR-30a-star	7.29	0.006488	hsa-miR-27b	3.26	0.003905
hsa-miR-195-star	7.2	0.002637	hsa-miR-1275	2.82	0.03935
hsa-miR-652	7.13	0.011589	hsa-miR-34a	2.82	0.015686

hsa-miR-324-3p	7.07	0.005221
hsa-miR-30a	7.02	0.004827
hsa-miR-297	6.92	0.001361
hsa-miR-378i	6.88	0.000432
hsa-miR-194	6.44	0.009015
hsa-miR-452	6.26	0.015139
hsa-miR-1247	6.22	0.002544
hsa-miR-505-star	6.09	0.004268
hsa-miR-4284	5.96	0.038169
hsa-miR-532-3p	5.87	0.000081
hsa-miR-299-5p	5.63	0.01047
hsa-miR-154	5.51	0.032924
hsa-miR-128	5.43	0.007011
hsa-miR-30c	5.34	0.001786
hsa-miR-4269	5.26	0.000167
hsa-miR-4793-3p	5.17	0.02202
hsa-miR-34c-5p	4.96	0.001871
hsa-miR-331-3p	4.9	0.00067
hsa-miR-1225-5p	4.77	0.034007
hsa-miR-362-5p	4.72	0.04484
hsa-miR-3197	4.45	0.041619
hsa-miR-30b	4.43	0.004626
hsa-miR-501-3p	4.35	0.030781
hsa-miR-425-star	4.16	0.000475
hsa-miR-127-5p	4.13	0.001932
hsa-miR-30d	4.11	0.000006
hsa-miR-345	3.98	0.021275
hsa-miR-654-3p	3.67	0.030187
hsa-miR-99b-star	3.64	0.01819
hsa-miR-17-star	3.5	0.000944
hsa-miR-34c-3p	3.48	0.007908

hsa-miR-4298	2.65	0.002452
hsa-miR-4721	2.45	0.024588
hsa-miR-3178	2.38	0.037049
hsa-miR-100	2.15	0.000079
hsa-miR-3141	2.06	0.029206
hsa-miR-23b	2.04	0.030062

hsa-miR-378d	3.42	0.017776
hsa-miR-130a	3.32	0.004766
hsa-miR-342-3p	3.3	0.002155
hsa-miR-3687	3.25	0.019111
hsa-miR-193a-3p	3.17	0.033552
hsa-miR-4530	3.16	0.012724
hsa-miR-4436b-5p	3.06	0.012413
hsa-miR-1202	3.01	0.019965
hsa-miR-940	2.99	0.024743
hsa-miR-4462	2.98	0.03002
hsa-miR-29b-2-star	2.91	0.02943
hsa-miR-4417	2.89	0.017638
hsa-miR-2110	2.86	0.002066
hsa-miR-887	2.86	0.00653
hsa-miR-4701-3p	2.84	0.002339
hsa-miR-197	2.8	0.027679
hsa-miR-1287	2.74	0.024152
hsa-miR-484	2.7	0.035617
hsa-miR-93-star	2.6	0.018311
hsa-miR-152	2.54	0.00106
hsa-miR-199b-3p	2.45	0.002804
hsa-miR-4539	2.43	0.021657
hsa-miR-574-3p	2.42	0.002256
hsa-miR-199a-3p	2.38	0.00229
hsa-miR-25	2.32	0.004384
hsa-miR-28-3p	2.29	0.000736
hsa-miR-4502	2.29	0.023208
hsa-miR-532-5p	2.27	0.04487
hsa-miR-629	2.24	0.031866
hsa-miR-4481	2.22	0.007365
hsa-miR-664-star	2.19	0.007651

hsa-miR-10a	2.11	0.04234
hsa-miR-3064-5p	2.1	0.01885
hsa-miR-3607-5p	2.1	0.002932
hsa-miR-487b	2.1	0.002432
hsa-miR-151b	2.06	0.000483

Day 3 versus day 28
Down regulated miRNAs

Transcript ID	Fold Change	ANOVA p-value
hsa-miR-324-3p	10.87	0.011269
hsa-miR-1275	5.83	0.010978
hsa-miR-99a	5.02	0.009488
hsa-miR-1268	4.89	0.047554
hsa-miR-195	4.21	0.019474
hsa-miR-4481	2.83	0.032049
hsa-miR-574-5p	2.5	0.005816
hsa-miR-155	2.34	0.019322
hsa-miR-409-3p	2.2	0.009787
hsa-miR-610	2.19	0.009868

Day 3 versus day 28
Up regulated miRNAs

Transcript ID	Fold Change	ANOVA p-value
hsa-miR-210	85.63	0.042315
hsa-miR-27b	27.04	0.048059
hsa-miR-181a-2-star	17.57	0.00042
hsa-miR-31-star	10.86	0.029896
hsa-miR-4721	7.34	0.014327
hsa-miR-34a	5.32	0.048953
hsa-miR-424	3.94	0.001373
hsa-miR-181a	3.53	0.0032
hsa-miR-424-star	2.96	0.015035
hsa-miR-23b	2.32	0.02683

Day 28 (passage 4) versus Day 63 (passage 9)
Down regulated miRNAs

Transcript ID	Fold Change	ANOVA p-value
hsa-miR-181a-2-star	3.83	0.014269
hsa-miR-4669	3.48	0.047324
hsa-miR-615-3p	3.22	0.039418
hsa-miR-28-3p	2.44	0.004675
hsa-miR-503	2.44	0.002095
hsa-miR-4298	2.3	0.025453

Day 28 (passage 4) versus Day 63 (passage 9)
Up regulated miRNAs

Transcript ID	Fold Change	ANOVA p-value
hsa-miR-3187-3p	41.65	0.034854
hsa-miR-3180-3p	12.95	0.029579
hsa-miR-3195	12.67	0.040444
hsa-miR-3937	10.92	0.023106
hsa-miR-3188	10.52	0.029434
hsa-miR-885-3p	10.47	0.002792

hsa-miR-424-star	2.28	0.00381
hsa-mir-503	2.23	0.015984
hsa-miR-28-5p	2.14	0.026066
hsa-miR-130b	2.08	0.041787

hsa-miR-1225-5p	9.8	0.024092
hsa-miR-572	8.43	0.025184
hsa-miR-3621	7.42	0.008952
hsa-miR-4690-5p	7.13	0.020401
hsa-miR-1909	6.88	0.019548
hsa-miR-3180	6.7	0.019376
hsa-miR-1910	6.38	0.03781
hsa-miR-4734	6.27	0.016938
hsa-miR-4655-5p	6.01	0.041629
hsa-mir-3656	5.91	0.000357
hsa-miR-4707-5p	5.85	0.012346
hsa-miR-4750	5.46	0.021968
hsa-mir-4634	5.3	0.031831
hsa-miR-4492	5.03	0.024152
hsa-miR-4721	4.98	0.042674
hsa-mir-3180-3	4.87	0.004868
hsa-miR-4763-3p	4.65	0.005639
hsa-miR-3648	4.6	0.002258
hsa-mir-4449	4.41	0.04596
hsa-miR-4741	4.32	0.045408
hsa-miR-4651	4.3	0.007714
hsa-mir-4734	4.26	0.009167
hsa-miR-149-star	4.24	0.005836
hsa-miR-3178	4.21	0.00649
hsa-miR-1228-star	4.15	0.001435
hsa-miR-3185	4.15	0.01492
hsa-miR-3656	4.11	0.00384
hsa-miR-4758-5p	3.96	0.024649
hsa-miR-718	3.9	0.019622
hsa-mir-3180-1	3.83	0.002869
hsa-mir-3180-2	3.83	0.024929

hsa-miR-1915	3.74	0.017185
hsa-miR-4467	3.71	0.007232
hsa-miR-3940-5p	3.61	0.014529
hsa-miR-4466	3.57	0.011763
hsa-miR-4745-5p	3.54	0.02597
hsa-miR-4508	3.53	0.010064
hsa-miR-3665	3.48	0.024706
hsa-miR-4530	3.44	0.003896
hsa-miR-92b-star	3.44	0.03693
hsa-miR-4488	3.39	0.007019
hsa-miR-638	3.37	0.011849
hsa-miR-3196	3.33	0.006577
hsa-miR-4516	3.33	0.034687
hsa-miR-328	3.32	0.041762
hsa-miR-4649-5p	3.31	0.045824
hsa-miR-2861	3.14	0.009969
hsa-miR-663	3.14	0.010947
hsa-miR-4497	3.12	0.002315
hsa-miR-1908	3.02	0.014638
hsa-miR-4505	3.02	0.037557
hsa-miR-4787-5p	2.98	0.022609
hsa-mir-4466	2.96	0.012654
hsa-miR-1268	2.94	0.025352
hsa-miR-1469	2.91	0.005998
hsa-mir-3648	2.9	0.009327
hsa-miR-762	2.89	0.014819
hsa-miR-4507	2.86	0.039236
hsa-miR-3960	2.84	0.007387
hsa-miR-4739	2.8	0.010407
hsa-mir-4523	2.71	0.014745
hsa-miR-4532	2.47	0.012785

hsa-miR-4665-5p	2.44	0.046849
hsa-mir-4758	2.34	0.024037
hsa-mir-3917	2.28	0.001473
hsa-mir-4469	2.22	0.001452
hsa-miR-532-3p	2.17	0.005698
hsa-mir-4508	2.16	0.026541
hsa-mir-4792	2.11	0.002061
hsa-mir-4787	2.01	0.001007

Supplemental Table S2- The proliferation of cultured cells reduces over time.

Passage number	Slope of growth line
P2	0.1023 ± 0.009177
P6	0.06997 ± 0.004195
P13	0.05987 ± 0.005494
P19	0.06393 ± 0.007630
P25	0.08339 ± 0.008922
P31	0.02863 ± 0.004826

ASC were grown continuously from passage 2 to passage 31 in standard media with a 1:1 dilution when cell confluence reached approximately 80 %. Their proliferation potential was assessed using the CellTiter 96® AQueous Non-Radioactive Cell Proliferation Assay Kit (Promega), according to manufacturers instructions. In brief, at each passage shown, cells were seeded in triplicate in a 96-well plate and subjected to the CellTitre assay at day 0, day 3 and day 7. Absorbance readings were taken, averaged and data points were graphed and analysed used linear regression. The slope of the growth line is shown. A higher number represents a steeper
